# Juglans regia *L*. (Walnut) Leaf Extract Ameliorates Pulmonary Edema Against Airway Inflammation via Upregulation of Tight Junction Proteins and Heme Oxygenase-1 in the Lungs of Asthmatic Mice

**DOI:** 10.1155/jotm/6976932

**Published:** 2025-05-19

**Authors:** Neelam Arshed, Arham Shabbir, Humaira Majeed Khan, Tabinda Fatima, Esraa M. Haji, Farhan K. Alswailmi, Ali F. Almutairy, Ashfaq Ahmad

**Affiliations:** ^1^Department of Pharmacology, Institute of Pharmacy, Faculty of Pharmaceutical and Allied Health Sciences, Lahore College for Women University, Jail Road, Lahore, Pakistan; ^2^Department of Pharmaceutical Chemistry, College of Pharmacy, University of Hafr Al Batin, Hafr Al Batin, Saudi Arabia; ^3^Department of Pharmacy Practice, College of Pharmacy, University of Hafr Al Batin, Hafr Al Batin, Saudi Arabia; ^4^Department of Pharmacology and Toxicology, College of Pharmacy, Qassim University, Buraydah, Saudi Arabia

**Keywords:** allergic asthma, inflammation, *Juglans regia .L.*, tight junction protein, Zonula occludens-1 (ZO-1)

## Abstract

**Background:** *Juglans regia* L. is renowned for its traditional use as a cure for respiratory diseases such as asthma and sinusitis.

**Objectives:** This study was intended to assess the protective mechanism of the effects of hydroalcoholic extract of the leaf of *Juglans regia* L. against airway inflammation and pulmonary edema by measuring the expression levels of heme oxygenase-1 (HO-1), occludin, and Zonula occludens-1 (ZO-1) in the lung tissues.

**Methods:** Ovalbumin (OVA) was used intraperitoneally to sensitize mice on Days 0 and 14 to induce allergic asthma by the intraperitoneal route. Animals were divided into 5 groups, consisting of normal control (NC), disease group (OVA, i.p), low-dose *J. regia* (LDJR) and high-dose *J. regia* (HDJR), methylprednisolone (MP), and reference control (RC) drug. On the 28^th^ day, blood and bronchial alveolar lavage fluid (BALF) were collected for total leukocyte (TLC) and differential leukocyte (DLC) analysis. Hematoxylin and eosin (H&E) and periodic acid–Schiff (PAS) staining of the lungs were performed for the architectural changes caused by OVA-induced bronchial asthma. Tight junction proteins were assessed by measuring the expression levels of HO-1, occludin, and ZO-1 in the lung tissues by using real-time polymerase chain reaction.

**Results:** Scores of inflammations, edema, and goblet cell hyperplasia were significantly increased (all *p* ≤ 0.05) in the DC group compared to the NC group, while treatment with LDJR and HDJR significantly reduced (all *p* ≤ 0.05) the scores of inflammations, edema, and goblet cell hyperplasia compared to the DC group. Real-time polymerase chain reaction data showed that expression levels of HO-1, occludin, and ZO-1 in lung tissues of the DC group were significantly reduced (all *p* ≤ 0.05), when the same was compared to the NC group, while treatment with LDJR and HDJR significantly increased (*p* ≤ 0.05) their expression level when compared to the DC group.

**Conclusion:** *Juglans regia* L.'s hydroalcoholic extract possesses antiasthmatic activity by normalizing the TLC cells and DLC cells. *Juglans regia* L.'s hydroalcoholic extract resulted in the amelioration of pulmonary edema which is attributed to the upregulation of HO-1, occludin, and ZO-1 in the lung tissues of the *Juglans regia L. treated* groups when compared to the diseased control group. Administration of *Juglans regia* L.'s extract also reduces the scores of inflammation and vascular congestion by evaluation of the lungs' histopathology in the disease control group when compared to the NC group.

## 1. Introduction

Asthma characterized by shortness of breath [1] and an allergic inflammatory disease causes the inflammation of the airway, lung tissue edema, and airway obstruction [2]. Many factors including environmental, microbiomes, and biodiversity of the mechanism are involved in the pathogenesis of asthma and allergic sensitization [3]. Leukocytes which include neutrophils, basophils, and eosinophils are collectively called as granulocytes, while monocytes and lymphocytes (T. cells and B. cells) are complex and important players of the immune system [4]. Circulating leukocytes are important markers of the immune response and are linked with the onset of diseases such as irritable bowel disease [5, 6]. Activated leukocytes are involved in the onset of airway inflammation by activating the mitogen-activated protein kinase pathway of inflammation [7]. Differential leukocyte (DLC) cells [8], eosinophils, neutrophils, lymphocytes, and mast cells [9, 10], are measured in ovalbumin (OVA)-induced bronchial asthma. Eosinophils are reported in the airway and bronchial alveolar lavage fluid (BALF) of asthmatic patients [11, 12], which causes allergic airway inflammation and pulmonary edema [13, 14]. Histopathological changes such as goblet cell hyperplasia (GCH), basement membrane thickening, and hypertrophy of the smooth muscle are due to the damage of the subepithelial and epithelial cells [15].

Pulmonary edema is also manifested by the presence of excessive fluid in the extravascular compartments of the lungs and is a result of the interruption of the paracellular alveolar barrier [16]. Tight junction (TJ) proteins are the primary reason for the regulation of the paracellular barrier. The movement of solutes, water, and immune cells is controlled by this paracellular barrier between both epithelial and endothelial cells [17]. These are heteromeric proteins and are vital for the exchange of materials within the lungs [18]. Transmembrane proteins such as occludins, claudins, and intracellular adapter proteins such as cingulin and Zonula occludens (ZO-1) constitute as the most important components of TJs in the alveolar epithelium [18]. Occludin is essential for maintaining the alveolar epithelial barrier [19], whereas ZO-1 is a binding protein serving as a link between the actin cytoskeleton and transmembrane TJ proteins. Apart from binding to cytoskeletal actin filaments, ZO-1 is involved in the regulation of the gene expression, where it forms a complex with the binding protein [20]. It has a pivotal role in maintaining the physiology of the alveolar epithelial barrier [21].

Any alteration in the assembly, relative expression, and localization of these proteins inside the TJ complexes causes the dysfunction of TJs and increased paracellular permeability [22]. Pulmonary edema is associated with the disturbance of paracellular alveolar barriers. Integrity of this barrier's function is very important, as any alteration in the barrier function can cause allergen sensitization and inflammation [23]. Prevention of the disruption of barriers and upregulation of these TJ proteins is helpful in the treatment of edema. Environmental factors such as proteases released from the dust mites and white blood cells, for example, eosinophils and neutrophils, also degrade ZO-1 and other adhesion junction (AJ) molecules [20]. Downregulation of these TJ and AJ molecules causes the activation of T helper Type 2 (Th2)–mediated pathways and contributes to the inflammatory cell recruitment [24]. Resultantly, fragility of the airway epithelium is compromised due to these proteases in asthma [25].

Heme oxygenase-1 (HO-1) is an isoform of heme oxygenase enzyme which is linked with asthma [26]. It is essential for protecting the lungs against inflammation [27] and injury caused by oxidative stress in cells and tissues [28]. In the lungs, HO-1 is present in different cell types, including Type II pneumocytes and alveolar macrophages. It is stimulated by hypoxia, nitric oxide (NO), and proinflammatory cytokines [29]. It has been observed that targeting the tumor necrosis factor–like ligand 1 A (TL1A) can be a therapeutic strategy in asthma [30]. Upregulation of HO-1 decreases airways inflammation, mucus secretion, and airways hyperresponsiveness to histamine [31]. The enzymatic reaction products released during the metabolism of heme are primarily responsible for its cytoprotective effects of HO-1. The induction of HO-1 and the delivery of these enzymatic products are mostly considered therapeutic approaches now a days [32].

Therapeutic strategy to manage asthma involves inhaled β2 agonists and corticosteroids, which have several local and systemic adverse effects [33–35]. These effects incline the patients and physicians to give more preference to medicinal plants and their constituents as a source of alternative therapies [36]. *Juglans regia* L. (*J. regia* L.) belongs to the Juglandaceae family and is commonly known as “walnut.” It widely grows in Asia and Europe. Different parts of this medicinal plant, such as the leaf, bark, seeds, leaf, and green husks, have been used as natural remedies in folk medicine [37]. The leaves of walnut have been extensively used in the treatment of skin inflammation [38], as anthelmintic, antioxidants [39, 40], hepatoprotective [41] antiseptic, antimicrobial [42], astringent, neuroprotective [43], cardiovascular diseases [44], and chemopreventive [45]. This plant has also been used as a cure for cardiorenal damage [46], diabetes, and asthma [47]. Certain studies revealed that the hydroalcoholic extract of *J. regia* L. possessed analgesic activities, anti-inflammatory activities [48], and wound healing properties with silver nanoparticles [49]. Previously, the kernel of *J. regia* L. has been shown to downregulate the proinflammatory cytokines in the asthmatic model [50]. The current study aimed to evaluate the effectiveness of the hydroalcoholic extract of *J. regia* leaves against lung tissue edema induced by OVA in mice. Furthermore, the present study set out with the objectives to measure the mRNA expression levels of HO-1 enzyme, occludin, and TJ proteins (ZO-1) in the lung tissue of the normal and asthmatic mice before and after the treatment with *Juglans by using real- time PCR.* Histopathological investigation was performed to observe the architectural changes in the airway inflammation, pulmonary edema, and vascular congestion in the lungs by using hematoxylin and eosin (H&E) staining along with the periodic acid–Schiff (PAS) staining.

## 2. Materials and Methods

### 2.1. Collection of Plant and Preparation of the Extract

*J. regia* leaves were obtained from Jinnah Garden, Lahore, in the month of November of 2023. The plant was identified by Dr Zaheer-ud-Din Khan, Department of Botany, Govt. College University Lahore. The specimen was also kept in the herbarium of the said department with herbarium number GC.Herb.Bot.3726. The adulterants were removed, and the leaves were dried under shade for 12 days. After this process, leaves were ground in a miller to get coarse powder. The powder obtained after grinding was passed through a mesh sieve no. 40 and weighed. The extract was prepared using previously published methodology [48, 51]. Hydroalcoholic extract 70%–30% was prepared, powdered, and macerated at room temperature for 7 days. Filtration was performed after 7 days of maceration by using a muslin cloth and Whatman No. 1 filter paper. The extracted material was collected and concentrated on a rotary evaporator (40°C). The concentrated extract was stored at 4°C in a refrigerator [52]. The extract was dissolved in normal saline (NS) just before administration to animals. The percentage yield of the extract was found as 15%.

### 2.2. Experimental Animals

A total number of 30 male mice (28–32 g) were purchased from the University of Veterinary & Animal Sciences, Lahore. All the mice were kept at standard room temperature (22°C–24°C) and humidity (45%–65%) conditions in the animal house of the University of Veterinary & Animal Sciences, Lahore, under a 12 h natural light/dark cycle. Standard pallet diet and water *ad libitum* were provided to the mice [53, 54]. For the experimental protocol, approval was sought from the Ethical Research Committee, University of Veterinary & Animal Science, Lahore (IREC 413).

### 2.3. Induction of Allergic Airway Inflammation and Edema

The experimental model of allergic airway inflammation and edema was induced in the mice by a previously reported study [55]. In brief, a weight of 20 μg of OVA (Sigma Aldrich, USA) dissolved in 2 mg of aluminum sulfate in 0.1 mL phosphate buffer saline (adjuvant) was used to develop allergic asthma in mice. On Days 0 and 14, initial sensitization of all groups was performed by intraperitoneal administration of 20 μg of OVA, except the normal control (NC) group. On Day 21, mice were given OVA again (1 mg/mL PBS) through the intranasal route for seven consecutive days (i.e., Days 21–27). PBS solution was used for the sensitization and challenge of the NC group [55].

### 2.4. Experimental Design

**Figure 1:**
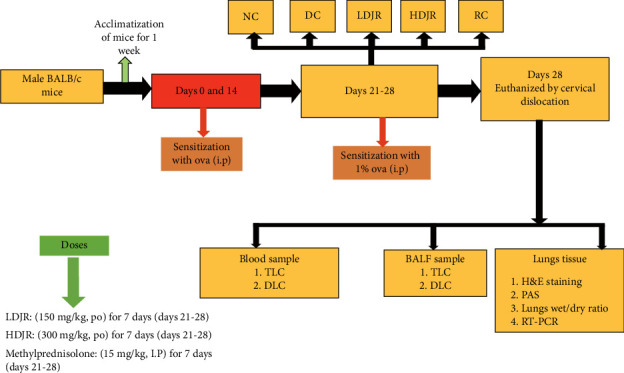
Flow sheet diagram of the experimental design to evaluate the role of *Juglans regia* L.'s (walnut) leaf extract to ameliorate pulmonary edema against airway inflammation.

A total number of 30 healthy mice were taken for experimental purposes and distributed into 5 groups of 6 mice each (*n* = 6). Doses were selected from previously reported data [48]. Animals were divided into the following groups: (1) NC group, which received NS for one week from Days 21–27; (2) disease control (DC) group, which received OVA by intraperitoneal route for sensitization on Days 0 and 14; (3) low-dose treated group (low-dose *J. regi*a [LDJR]) mice, which were treated with the low dose of *J. regia* L. leaves' hydroalcoholic extract (150 mg/kg b.w.) pretreated with OVA (I.p) on Days 0 and 14; (4) high-dose treated group (high-dose *J. regia* [HDJR]) mice, which were treated with the high dose of *J. regia* L.'s leaves hydroalcoholic extract (300 mg/kg b.w.) pretreated with OVA (I.p) on Days 0 and 14. Doses were administered with the help of gavage for 7 days (i.e., Days 21–27); and (5) the reference drug–treated group (reference control [RC]), which was treated with methylprednisolone (MP) (15 mg/kg b.w) was used as the reference drug and intraperitoneally administered to this group for 7 days [56] pretreated with OVA (I.p) on Days 0 and 14. On the 28^th^ day, after the last challenge and treatment, blood samples were drawn and evaluated for total blood count cells by using an automated hematology analyzer. Later, mice were euthanized by cervical dislocation to collect the lung tissue for different parameters related to lungs and histopathology as shown in Figure 1.

Collection of BALF from the mice of NC, DC, and treatment groups.

BALF was collected by using a method reported in [57]. In brief, mice were placed on a surgical table and were fixed on it by pinning down the limbs. After disinfecting the neck, the trachea was exposed, and access was made to the sternohyoid muscle by separating the salivary glands. A cotton thread was placed around the trachea. A propylene cannula was placed inside the trachea at 0.5 cm depth to avoid deep penetration into the trachea and rupturing of the trachea. 1 mL of the balanced salt solution with 100 μM EDTA was loaded into the lungs by using a 1 mL syringe, and the solution was aspirated gently by pushing the catheter a little deeper into the trachea to facilitate the aspiration. Syringes were removed from the needle and the recovered lavage fluid was transferred to a tube of 15 mL volume already placed on ice.

### 2.5. Histopathological Evaluation Using H&E and PAS Staining

Histopathology was performed by following a study reported in [58]. One lobe of the dissected lung was taken and fixed in 10% neutral buffered formalin. Graded concentrations of ethanol were used for dehydration of the lung tissue. After fixation in paraffin wax, 5 mm-thick sections were cut using a microtome and slides were prepared. For evaluation of inflammatory cell infiltration, edema, and airway congestion, H&E staining was performed. For the identification of GCH, PAS staining was performed. The slides were examined in a blinded fashion by a histopathologist, and semiquantification of the results was performed by using the scoring scale as given in the previous publications (0, none; 1, mild; 2, moderate; and 3, severe) [56].

### 2.6. Lung Wet/Dry Weight Ratio

The lung wet/dry weight ratio is an index of lung tissue edema. One lobe of lung tissue was removed and weighed immediately. Then, the same lobe was oven dried at 56°C for 15 min and weighed again [59].

### 2.7. Determination of mRNA Expression Levels of Occludin, ZO-1, and HO-1

RT-qPCR technique was used to measure the mRNA expression levels of occludin, ZO-1, and HO-1 [60]. The standard TRIzol method was used for isolation of total RNA from lung tissue [61], and the methodology for quantification of mRNA quantification was adopted as reported in [62]. To determine the yield of total RNA, the NanoDrop spectrophotometer was used [63]. A weight of 1000 ng/reaction of total RNA was used for cDNA synthesis, and reverse transcription was performed as per the instructions mentioned in the kit (Revert Aid First Strand DNA synthesis Kit, Thermo Scientific). The primer sequences for occludin, ZO-1, and HO-1 are shown in Table 1.

Real-time PCR was performed by mixing 1 μL of cDNA sample, 12.5 μL of PCR master mix, 0.5 μl of each forward and reverse primer, and nuclease-free water q.s. To 25 μL. Rotor-Gene Q (QIAGEN, USA) thermal cycler was set for 45 cycles of denaturation (95°C for 15 s), annealing (60°C for 20 s), and extension (72°C for 30 s). The Livak method 2^−(ΔΔCt)^ was applied for calculating the relative gene expression as reported [54], and target gene expression was normalized to GAPDH expression [60].

### 2.8. Statistical Analysis

Statistical analysis was performed by using GraphPad Prism (Version 9) software. Mean ± standard deviation was used to express all results. One-way analysis of variance (ANOVA) followed by post hoc Tukey's test was used for data analysis and to check significant differences among groups. A *p* value of ≤ 0.05 was considered as statistically significant. ∗ Denotes the comparison of treatment groups with the DC group, while # denotes the comparison of the DC group with the NC group.

## 3. Results

### 3.1. Effects of *J. regia* L.'s Extract on Total Leukocyte (TLC) Count and Neutrophil, Eosinophil, Lymphocyte, and Monocyte Counts in the Blood

**Figure 2:**
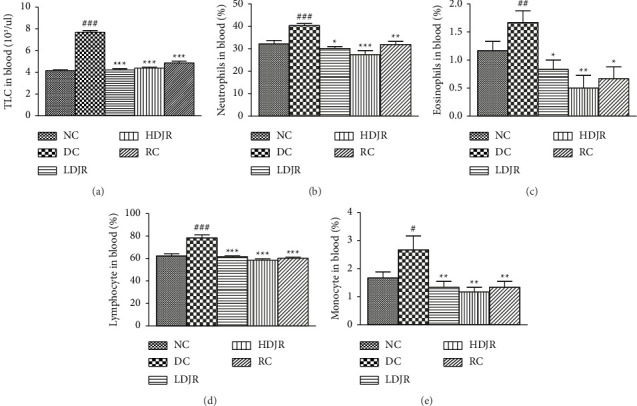
Effects of different doses of *J. regia*, i.e., LDJR (150 mg/kg) and HDJR groups (300 mg/kg), on TLC and DLC in blood. Data are given as ± SEM. ^#^(*p* ≤ 0.05) versus normal control (NC) and ^∗^(*p* ≤ 0.05) versus disease control (DC).

A significant increase in TLC counts of the disease group (8 ± 0.4) was observed as compared to the NC group (4 ± 0.2). Treatment with *J. regia* L. in both low-dose (4. ± 0.3) and high-dose (4 ± 0.2) groups and MP (5 ± 0.4) attenuated TLC in blood significantly (Figure 2(a)). The neutrophil count in the blood of the DC group (40 ± 2) was increased as compared to the NC group (32.17 ± 3.869). Treatment with *J. regia* L. at low-dose (34 ± 3.0), high-dose (27 ± 4.5), and MP (31 ± 3) attenuated the neutrophil counts in blood significantly (Figure 2(b)). Eosinophil counts in the DC group (1.7 ± 0.5) were raised as compared to the NC group (1.1 ± 0.4). Treatment with *J. regia* at low-dose (0.8 ± 0.4), high-dose (0.5 ± 0.5), and MP (0.7 ± 0.5) attenuated the levels of eosinophils significantly (Figure 2(c)).

Lymphocyte counts in the DC group (79 ± 7) were increased as compared to the NC group (63 ± 5). Treatment with *J. regia,* both at low dose (62 ± 3) and high dose (59 ± 2), decreased the levels of lymphocytes. Treatment with the reference drug MP (60 ± 3) also attenuated the levels of lymphocytes significantly when compared with the disease group (Figure 2(d)). Monocyte counts in the DC group (2.5 ± 0.5) (2.7 ± 1.2) were raised as compared to the NC group (1.50 ± 0.5). Treatment with *J. regia* L. at low dose (1.3 ± 0.5) and high dose (1.1 ± 0.4) attenuated the levels of monocytes significantly (Figure 2(e)).

### 3.2. Effects of *J. regia* L.'s Extract on TLC Count and Neutrophil, Eosinophil, Lymphocyte, and Monocyte Counts in the BALF

**Figure 3:**
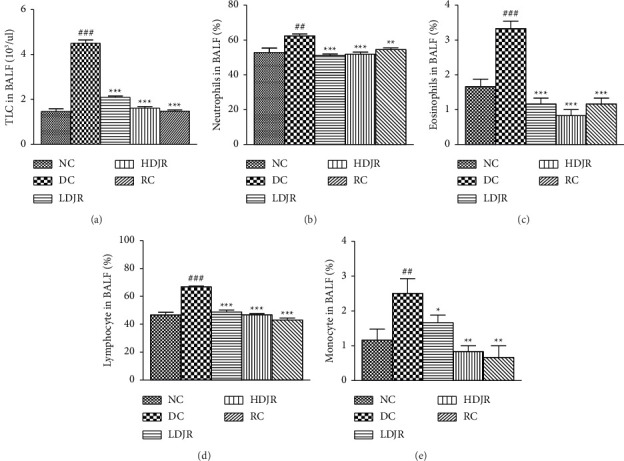
Effects of different doses of *J. regia*, i.e., LDJR (150 mg/kg) and HDJR groups (300 mg/kg), on TLC and DLC in BALF. Data are given as ± SEM. #(*P* ≤ 0.05) versus normal control (NC) and ^∗^(*P* ≤ 0.05) versus disease control (DC).

A significant increase in TLC of the DC group (10^3^/μL) (4.50 ± 0.37) was observed compared to the NC group (1.5 ± 0.3). Treatment with *J. regia* significantly attenuated TLC in BALF at both doses (Figure 3(a)). Neutrophils (%) (62 ± 3.07), eosinophils (%) (3.5 ± 0.5), lymphocytes (%) (67 ± 2.2), and monocytes (%) (2.5 ± 1.0) were increased in DC groups as compared to NC groups (52.8 ± 6; 1.7 ± 0.5; 46.7 ± 4.7; and 1.2 ± 0.8, respectively). The data revealed that the number of neutrophils, eosinophils, lymphocytes, and monocytes (%) was decreased in the LDJR group (52 ± 3; 1.3 ± 0.5; 49 ± 3.6; and 1.17 ± 0.4, respectively). Neutrophils, eosinophils, lymphocytes, and monocytes (%) were also found reduced in the HDJR group (50.5 ± 3.7; 1.17 ± 0.7; 45.8 ± 3.2; and 0.83 ± 0.40, respectively) and the RC group (54 ± 2.1; 43 ± 3.6; and 0.7 ± 0.8, respectively) (Figures 3(b), 3(c), 3(d), and 3(e)).

### 3.3. Effects of *J. regia* L. on Histopathological Changes

#### 3.3.1. *J. regia* L. Significantly Decreased Airways Inflammation

**Figure 4:**
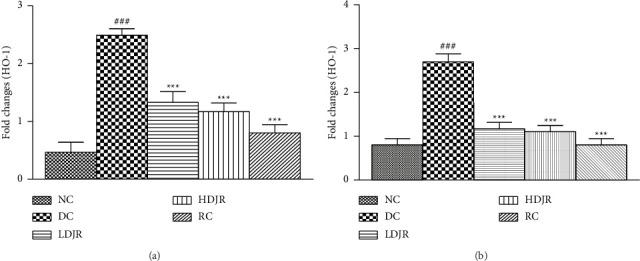
(a-b) Treatment with *J. regia* significantly attenuated inflammation (a) and edema and vascular congestion score (b). Data are given as ± SEM. ^#^(*p* ≤ 0.05) versus normal control (NC) and ^∗^(*p* ≤ 0.05) versus disease control (DC).

**Figure 5:**
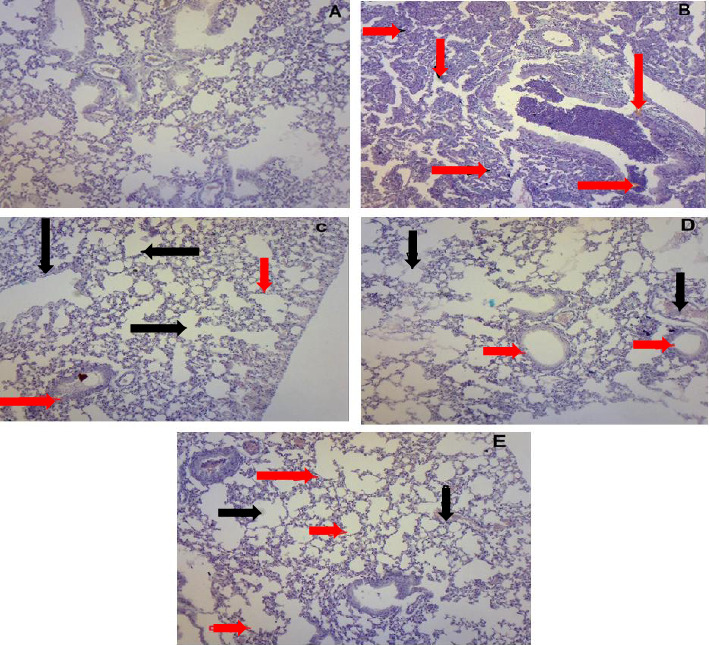
(A-E) H&E staining; *Juglans regia* L. *extract* attenuated the inflammation and vascular congestion in lung airways. The lung section of the NC group mice represented by image (A) shows no inflammation. The lung section of the DC group is shown in image (B). Inflammation in airways is shown with red arrows, while edema and congestion are shown with black arrows in the DC group (B). In (C–E), red arrows show a reduction in inflammation, whereas black arrows indicate the resolution of edema and vascular congestion after treatment with *J. regia* extract, 150 and 300 mg/kg, and methylprednisolone, respectively.

Infiltration of inflammatory cells in airways was observed in the lungs of the DC group (2.83 ± 0.408) upon exposure to OVA. The lungs of the mice in the NC group are normal with no obvious inflammatory cells. *J. regia* L.'s extract significantly decreased inflammatory cells in the LDJR (1.33 ± 0.5), HDJR (1.17 ± 0.4), and RC groups (0.8 ± 0.4) with *p* value < 0.001 (Figures 4(a), 4(b), 5(A), 5(B), 5(C), 5(D), and 5(E)).

#### 3.3.2. *J. regia* L. *Significantly* Inhibited Edema and Vascular Congestion

Edema and vascular congestion were observed in the lungs of the DC group (2.7 ± 0.5) when compared to the NC group (0.83 ± 0.40) (*p* value < 0.001). Treatment with *J. regia* L. (1.17 ± 0.40) and reference drug MP (0.83 ± 0.40) significantly decreased edema and vascular congestion as compared to the disease group (*p* value < 0.001) (Figures 4(b), 5(A), 5(B), 5(C), 5(D), and 5(E)).

#### 3.3.3. *J. regia* L. Significantly Alleviated GCH

**Figure 6:**
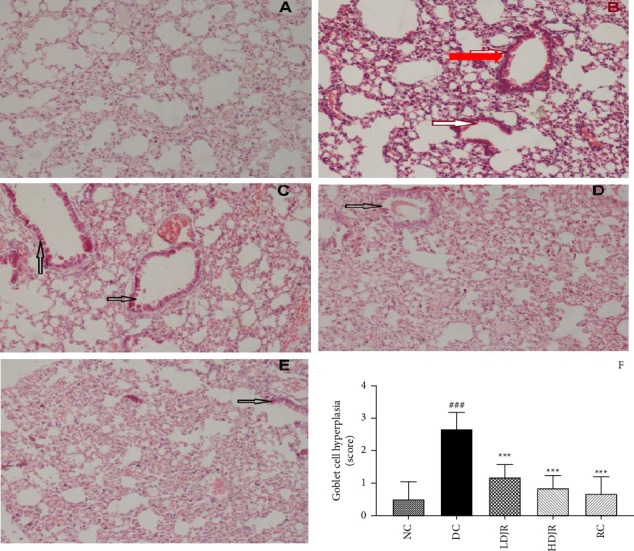
(A-F) PAS staining; *J. regia* extract reduced goblet cells in lung airways. The long section of the control group mice is represented by image (A). Red arrows show hyperplasia of goblet cells in the DC group (B). Treatment with LDJR, HDJR, and MP decreased the goblet cell hyperplasia as shown by black arrows in image (C–E), respectively. Data are given as ± SEM. ^#^(*p* ≤ 0.05) versus normal control (NC) and ^∗^(*p* ≤ 0.05) versus disease control (DC). Figure 6(F).

A significant hyperplasia of mucous-producing goblet cells was observed in the lungs of the DC group (2.7 ± 0.52) upon exposure to OVA. The lungs of mice in the NC group (0.50 ± 0.55) are normal with no obvious changes. *J. regia* L.'s extract significantly decreased goblet cells in the LDJR (1.167 ± 0.40), HDJR (0.83 ± 0.40), and RC groups (0.67 ± 0.52) with *p* value < 0.001 as shown in Figures 6(A), 6(B), 6(C), 6(D), and 6(E).

### 3.4. Effect of *J. regia* L. on Lung's Wet/Dry Weight Ratio

**Figure 7:**
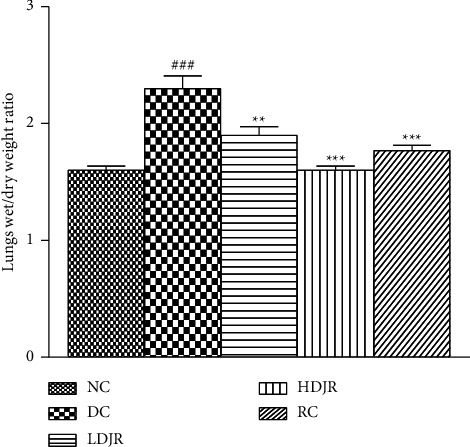
Effect of different doses of *Juglans regia* L. on lung wet/dry weight ratio. Pulmonary edema decreased significantly after treatment with the extract as compared to the DC group. Data are given as ± SEM. ^#^(*p* ≤ 0.05) versus normal control (NC) and ^∗^(*p* ≤ 0.05) versus disease control (DC).

The lung wet/dry weight ratio has been used as an indication of lung tissue edema. Data showed a significant increase in the lung wet/dry weight ratio of the DC group (2.3 ± 0.3) as compared to the NC group (1.6 ± 0.1). After treatment with *J. regia* L., the ratio was decreased in the low-dose group (1.9 ± 0.2), the high-dose group (1.6 ± 0.1), and the reference group (1.773 ± 0.128) (Figure 7).

### 3.5. Effects of *J. regia* L. on the Expression Levels of HO-1, Occludin, and ZO-1 in Lung Tissues

**Figure 8:**
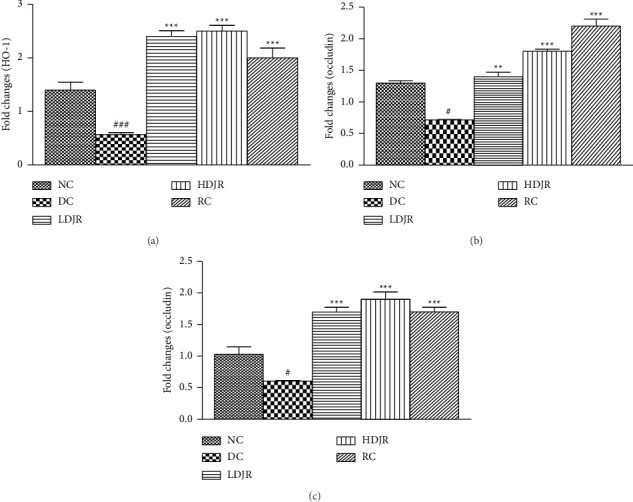
*Juglans regia* L. and MP significantly increased the mRNA expression levels of HO-1 and tight junction proteins when compared to the DC group. The mRNA expression levels were determined using the reverse transcription real-time PCR technique. Data are given as ± SEM. ^#^(*p* ≤ 0.05) versus normal control (NC) and ^∗^(*p* ≤ 0.05) versus disease control (DC).

The heme oxygenase plays an important role in protecting the lungs from inflammatory damage via preventing disruption of TJ proteins. Results demonstrated that mRNA expression of HO-1 decreased in the DC group (0.57 ± 0.1) as compared to the NC group (1.4 ± 0.4). Treatment with *J. regia* L.'s extract (2.4 ± 0.3) and MP (2.0 ± 0.5) significantly upregulated the mRNA expression of HO-1 as compared to the DC group (Figure 8(a)).

A significant downregulation of mRNA expression levels of occludin in the DC group (0.728 ± 0.08) was observed as compared to the NC group (1.3 ± 0.1). Treatment with *J. regia* in both low-dose (1.4 ± 0.2) and high-dose (1.8 ± 0.1) groups and MP (2.2 ± 0.3) increased the mRNA expression levels of occludin in lung tissues significantly (Figure 8(b)).

The expression level of ZO-1 in lung tissues of the DC group (0.6 ± 0.04) was decreased compared to the NC group (1.04 ± 0.3). Treatment with *J*. *regia* extract in both low-dose (1.7 ± 0.2) and high-dose (1.9 ± 0.3) groups and MP (1.7 ± 0.2) increased the expression level of ZO-1 in lung tissues significantly (Figure 8(c)).

## 4. Discussion

The current study aimed to evaluate the effectiveness of the hydroalcoholic extract of *J. regia* leaves against lung tissue edema induced by OVA in mice. Second, the present study set out with the objectives to measure the mRNA expression levels of HO-1 enzyme, occludin, and TJ proteins, particularly ZO-1, in the lung tissue of the normal and asthmatic mice before and after the treatment with *Juglans* by using real-time PCR. Lastly, histopathological investigation was performed to observe the architectural changes in the airway inflammation, pulmonary edema, and vascular congestion in the lungs by using H&E staining along with the PAS staining. The present study came up with the findings that *J. regia* L.'s hydroalcoholic extract possesses antiasthmatic activity by normalizing the TLC cells and DLC cells. Second, *J. regia* L.'s *hydroalcoholic* extract resulted in the amelioration of pulmonary edema, which is attributed to the upregulation of HO-1, occludin, and ZO-1 in the lung tissues of *J. regia* L– treated groups when compared to the DC group. Lastly, the administration of *J. regia* L.'s extract also reduced the scores of inflammation, edema, and vascular congestion by evaluation of lung histopathology of the DC group when compared to the NC group.

The present study showed a significant increase (*p* ≤ 0.05) in TLC count, neutrophils, eosinophils, lymphocytes, and monocytes in the blood (Figures 2(a), 2(b), 2(c), 2(d), and 2(e)) and BALF samples (Figures 3(a), 3(b), 3(c), 3(d), and 3(e)) in the DC group when compared to the NC group. This increased count is a characteristic feature of eosinophilic asthma and is linked to Th2–mediated response and allergic sensitization [64]. Treatment with *J. regia* L.'s extract significantly decreased (*P* ≤ 0.05) TLC count, neutrophils, eosinophils, lymphocytes, and monocytes in the blood (Figures 2(a), 2(b), 2(c), 2(d), and 2(e)) and BALF samples (Figures 3(a), 3(b), 3(c), 3(d), and 3(e)) in the DC group when compared to the NC group. These findings are consistent with the inferences of a previous study [65], where black seed oil was administered to suppress the OVA-induced lung injury. This study was also in line with a previously reported study, which reported the amelioration of the allergic bronchial asthma by *J. regia* L. by suppressing the proinflammatory cytokines by using aquaporin 1 and 5 [66], while antiasthmatic activity was also ameliorated by ethyl acetate and n-hexane fractions of *J. regia* kernel [50]. We previously reported that leukocyte infiltration is increased in the blood and sputum of the asthmatic patient [67], which are prooxidant in asthmatic patients [68]. The present study came up with the findings that administration of *J. regia* L.'s extract reduced the TLC and DLC in the blood and ameliorates their infiltration in the lungs which can be seen in the data obtained from the BALF samples (Figures 3(a), 3(b), 3(c), 3(d), and 3(e)). This will further reduce the inflammatory cytokines as reported in the earlier reported data [66]. It can be deduced that hydroalcoholic administrations of *J. regia* L.'s extract reduced the TLC and DLC in the blood and ameliorated their infiltration in the lungs and provided relief in bronchial asthma in the mice.

Histopathological findings during the current study demonstrated significant infiltration of inflammatory cells and edema with vascular congestion in the DC group as compared to the NC group (Figures 4(a) and 4(b)), and structural changes can be seen in Figures 5(A), 5(B), 5(C), 5(D), and 5(E). These outcomes are consistent with the inferences of previous study [69]. Alveolar thickening and edema formation are characteristic features of this disease and any inflammatory and structural damage to the airway wall can cause these histopathological changes [64]. Treatment with *J. regia* L.'s extract normalized the inflammation and edema scores in the lungs.

In this study, we also evaluated GCH, a characteristic pathological feature of bronchial asthma. Narrowing of airways and airflow obstruction are due to hypersecretion of mucus associated with GCH [70]. Hypersecretion of mucus associated with GCH and its reversal are represented by arrows in Figures 6(A), 6(B), 6(C), 6(D), 6(E), and 6(F). In this study, a significant increase in GCH was seen in DC groups, which can be seen by its score (Figure 6(F)). Treatment with *J. regia* L. significantly decreased the GCH at both doses when compared to the DC group.

Pulmonary edema is commonly determined by using the lung's wet/dry weight ratio [69]. In this study, a considerable increase in the lung wet/dry weight ratio was seen in the DC group after exposure to OVA allergen as shown in Figure 7. Treatment with *J. regia* L. decreased the ratio which suggests the effectiveness of the extract against pulmonary edema. We further evaluated its effectiveness against pulmonary edema by checking the mRNA expression level of HO-1 enzyme, occludin, and TJ proteins (ZO-1) in the lung tissue of the normal and asthmatic mice before and after the treatment with *J. regia* L. by using real-time *PCR*.

The present study explored that expression levels of HO-1 in the lung tissue of the bronchial asthma diseased group are reduced when compared to the NC group, while treatment with *J. regia* L. upregulates its expression as shown in Figure 8(a). HO-1 is a rate-limiting enzyme and is present in nearly every tissue in the body. It plays a defensive role in oxidative stress and inflammatory processes in the lungs [71]. Its exact mode of action is still not known, but some studies reported Urolithins A and B are the phytoconstituents responsible for antioxidant and anti-inflammatory activities of the J. regia *L*. [39]. Enzymatic reaction products of the enzyme, i.e., carbon monoxide, biliverdin, and iron, are responsible for the cytoprotective effects of HO-1 [32]. Recent studies suggested that an increase in HO-1 expression can improve airway inflammation and mucus hypersecretion, in an asthma model. Exogenous administration of HO-1 reaction products attenuates airway inflammation in the asthma model by inhibiting leukocyte migration [72]. An increase in leukocyte infiltration and decreased expression levels of HO-1 were observed in the DC group, which states the role of leukocytes and HO-1 in the pathophysiology of bronchial asthma. Hydroalcoholic extract of *J. regia* L. significantly upregulated the HO-1 levels in both treatment groups, which might have resulted in reduced leukocyte infiltration and inflammation in the treatment group. These results indicate that HO-1 shows anti-inflammatory effects by preventing the PMN migration into lung airways and are in line with previously reported studies [73].

HO-1 also plays a vital role in stabilizing the pulmonary barrier function. Induction of HO-1 is known to increase the expression level of ZO-1 and occludin [73]. It is plausible that the elevated expression levels of ZO-1 and occludin found in our study might be attributed to the upregulation of HO-1. The present study also describes the effect of *J. regia* L. on mRNA expression levels of occludin and ZO-1. OVA exposure considerably decreases the levels of occludin and ZO-1 in the DC group as shown in Figures 8(b) and 8(c). Previous studies have also shown the downregulation of TJ protein expression in the epithelium of asthmatic patients [74]. Treatment with *J. regia* L. upregulated the expression levels of these proteins, which suggests a reduction in pulmonary edema by preventing the disruption of the airway epithelium barrier and providing protection in the maintenance of lung architecture. Histopathological evaluation also showed the development of edema and vascular congestion in the DC group, which were ameliorated after treatment with *J. regia* L.'s leaf extract, and is in line with the data on the TJ proteins (ZO-1 and occludin).

## 5. Conclusion

The current study showed that *J. regia* L. possessed significant anti-inflammatory and antiedematous activities in OVA-induce mice model of allergic asthma. The data showed that *J. regia* L. attenuated the airway inflammation as shown by decreased infiltration of inflammatory cells in airways and TLC and DLC counts in both blood and BALF. *J. regia* L.'s hydroalcoholic extract resulted in the amelioration of pulmonary edema, which is attributed to the upregulation of HO-1, occludin, and ZO-1 in the lung tissues of the *J. regia* L.*–*treated groups when compared to the DC group.

## Figures and Tables

**Table 1:** Primer sequence along with product size.

Primers	Forward/reverse	Sequence	Product size
Occludin	Forward	5′-CCTCCAATGGCAAAGTGAAT-3′	243
	Reverse	5′-CTCCCCACCTGTCGTGTAGT-3′	

ZO-1	Forward	5′-GCCAGAGAAAAGTTGGCAAG-3′	226
	Reverse	5′-TTGGATACCACTGCGCATAA-3′	

HO-1	Forward	5′-CACGCATATACCCGCTACCT-3′	250
	Reverse	5′-AAGGCGGTCTTAGCCTCTTC-3′	

## Data Availability

The data that support the findings of this study are available on request from the corresponding author. The data are not publicly available due to privacy or ethical restrictions.
